# Public exposure to broadband electromagnetic fields and its association with population density and building density: The case study of Beijing^☆^

**DOI:** 10.1016/j.heliyon.2023.e17153

**Published:** 2023-06-20

**Authors:** Xinwei Song, Mengqi Han, Yijie Chen, Yuntao Yue

**Affiliations:** School of Electrical and Information Engineering, Beijing University of Civil Engineering and Architecture, Zhanlanguan Road 1, 100044, Beijing, China

**Keywords:** Electromagnetic field, Risk, Association rule, Population density, Building density

## Abstract

The gradual increase in electromagnetic field (EMF) exposure levels poses a potential threat to human health and the normal operation of electronic systems. In order to know the environmental EMF conditions, measurements were carried out on roads of about 400 km in the urban area of Beijing, China. The measurement results show that the electric field strength of about 89% of the sampling points is within 3 V/m, and the electric field strength of other sampling points is relatively high. Combined with further spectrum analysis, it was found that the electric field strength of one road section exceeded the national standard limits. In addition, to help quickly identify the general condition of the environmental EMF, a set of procedures for mining the association rules between the electric field strength and population density and building density is proposed in this paper. The final association rules show that the electric field strength is usually lower than 1.5 V/m in areas with medium or lower population density and areas with low building density; the electric field strength in areas with extremely high population density and areas with high building density is usually 1.5-4 V/m; while the electric field strength higher than 4 V/m mainly occurs in areas with extremely high population density. It is recommended to focus on strengthening the monitoring of EMF in areas with extremely high population density, and at the same time continuously pay attention to the trend of the urban EMF levels, so as to achieve early warning and treatment of relevant risks.

## 1 Introduction

With the development of wireless technologies, people are exposed to complex broadband electromagnetic fields (EMFs) due to various man-made radiation sources, such as radio and TV broadcast facilities, mobile phones and their base stations, radars, wireless induction and identification devices, etc. On the one hand, EMFs in the environment can induce undesired voltages and currents in electronic systems, which may lead to system failures [1]; on the other hand, they can cause thermal and non-thermal effects in the human body, which may induce diseases if exceed the suggested limits [2]. The above situation will be exacerbated with the increase of wireless applications, which has also led to public concern about EMF exposure. Therefore, to protect the public from hazards, many organizations and countries including China have set EMF controlling limits with reference to the recommended guidelines of the International Commission on Non-Ionizing Radiation Protection [3] (last revised in 2020 [4]).

In order to check whether the environmental EMF levels meet the local standards, many researchers have carried out measurements of EMF exposure, as in the literature in recent years [5], [6], [7], [8], [9]. To the best of the authors' knowledge, there are no cases of public exposure to broadband EMFs exceeding the controlling limits in the available literature. However, this study found an area where the electric field strength exceeded the limits during EMF monitoring on urban roads in Beijing. This should be paid attention to, as more man-made radiation sources or increasing transmission power due to urban development may result in more situations exceeding the controlling limits that cannot be detected in time.

However, it is impossible to monitor the environmental EMF in all areas at all time periods, even by multisensor networks [10], [11] or by drones coupled with measuring devices [12]. Then, is it possible to quickly identify the approximate level of the EMF through some information? Recently, [13] analyzed the correlation between man-made noise in several frequency bands and the number of houses within various radii in residential areas, and concluded that the correlation is strongest within a radius of 300 m. Inspired by this, we selected two types of information that are accessible and closely related to human activities, real-time population density and building density, to analyze their association with the environmental EMF. Due to the large volume of data and inconsistent data types, data mining [14] are used in this study, and a set of procedures for mining association rules between environmental EMF and real-time population density and building density is proposed. Data mining is a technique for discovering useful patterns and information in large data sets, among which association rule mining is used to discover meaningful connections hidden in data sets. Association rule mining has been applied in several fields such as learning material recommendation [15], geochemical data knowledge discovery [16], customer profiling [17], and traffic accident analysis [18], but there are few studies on its application for association analysis between environmental EMF and other data.

The rest of this paper first describes data acquisition methods of environmental EMF, population density and building density, which are the information of 13412 sampling points on about 400 km urban roads in Beijing. In Section 3, the results of public exposure to environmental EMF are shown, and the specific situation of the area where the electric field strength exceeded the controlling limits are presented. In Section 4, the method for the association analysis of environmental EMF, population density, and building density is introduced. In Section 5, the resulted association rules are given and discussed. Finally, the conclusion is drawn in Section 6. The purpose of this study is to point out the potential risks of public exposure to environmental EMF through the case of Beijing, and to provide a new way of quickly identify the status of the electromagnetic environment.

## 2 Data acquisition methods

### 2.1 Environmental EMF

The environmental EMF data are obtained by an electromagnetic radiation detector (Microrad PRO2). The instrument consists of a hand-held meter NHT 310 and a wideband electric field probe PROBE 01E which is isotropic. The measured frequency range is from 100 kHz to 6.5 GHz, the amplitude range is from 0.2 V/m to 350 V/m, and the uncertainty is ±5 dB. The electromagnetic radiation detector was set on a nonmetallic tripod on the roadside. The probe of the instrument was 1.7 m above the ground [19]. The measurement time of each sampling point was 6 min, so that the root mean square value over 6 min can be obtained [4].

**Figure 1:**
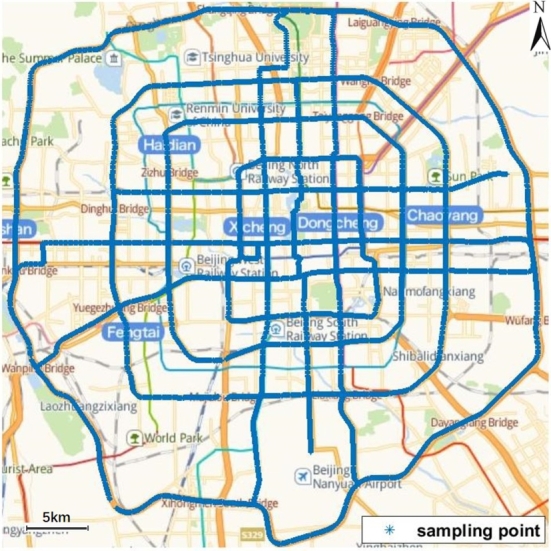
Sampling points for measurements displayed on the Beijing urban map.

Representative urban roads in Beijing were selected for sampling measurement, as shown in Fig. 1. The total length of the roads is about 400 km, and there are 13412 sampling points approximately evenly distributed with a spacing of about 30 m. This spacing is appropriately shortened or lengthened at places where pedestrians are not allowed to pass. The longitude and latitude coordinates of each sampling point were recorded while measuring the environmental EMF. The measurement campaign started at the beginning of 2021 and was conducted during 11:00-14:00 of the weekday, which is one of the peak periods of electromagnetic radiation in the urban environment.

### 2.2 Population density

**Figure 2:**
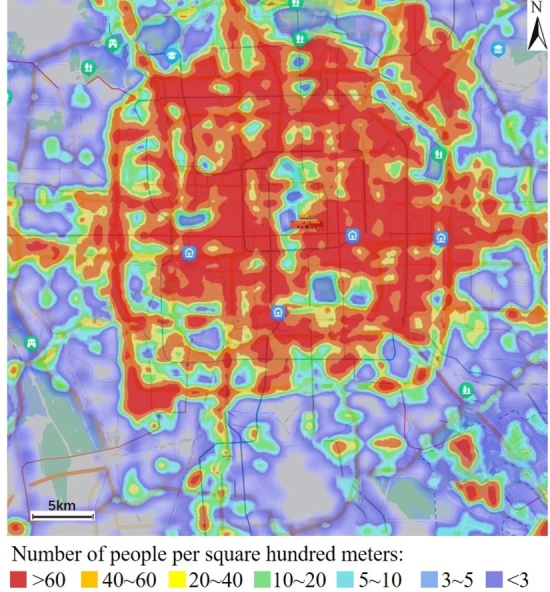
An example of the population density in Beijing urban area at a certain time.

Population density data can be obtained through some map software such as Baidu Map mobile application [20] or the software development kit (SDK) of Baidu Map for Andriod [21] or iOS [22]. The data is produced from geographic location information of millions of mobile phone users based on the location-based service (LBS) platform [23] operated by the Baidu Company in China. It is real-time data and has been used for population dynamics studies [24], [25], [26]. Fig. 2 is an example of the population density in Beijing urban area at a certain time. The population density is divided into 7 levels, represented in descending order by red, orange, yellow, green, blue, indigo, and purple. These colors corresponding to the number of people per square hundred meters, which are greater than 60, between 40 and 60, between 20 and 40, between 10 and 20, between 5 and 10, between 3 and 5, and less than 3, respectively. The population density data is updated every few minutes. The population density levels at sampling points were recorded while the environmental EMF was being measured. In most cases, the population density level did not change during the measurement of the EMF at a sampling point; if it did, the level that lasted longer was recorded.

### 2.3 Building density

**Figure 3:**
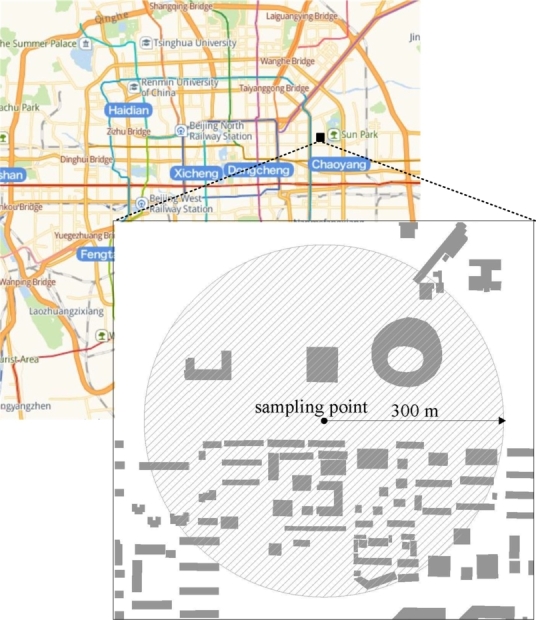
Schematic diagram of building density data interception.

Building density data can be obtained using geographic information system (GIS) software, such as ArcGIS used in this study. According to the research results of literature [13], the level of man-made electromagnetic noise has the strongest correlation with the number of houses within 300 m. Therefore, the total building area within a radius of 300 m with the sampling point as the center of the circle is used as the data to measure the building density in this study, as shown in Fig. 3. For the case of a large number of sampling points, the software can be controlled by a Python script for batch processing, so that the building density data corresponding to each sampling point can be obtained automatically by running the script.

## 3 Environmental EMF exposure analysis

**Figure 4:**
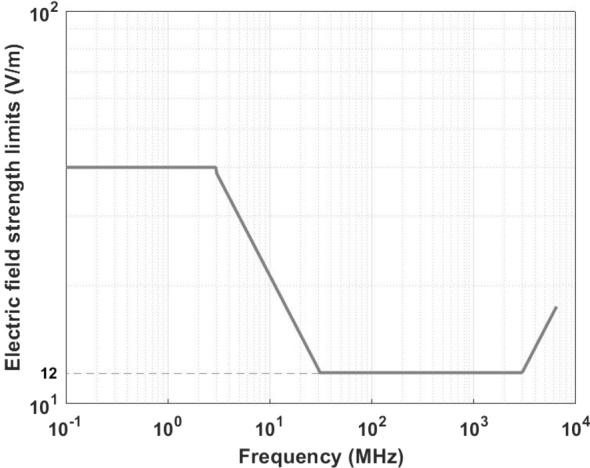
Electric field strength controlling limits from 100 kHz to 6.5 GHz.

The current standard in China for public exposure to environmental EMFs is GB 8702-2014 Controlling Limits for Electromagnetic Environment [27], which refers to [3]. The specific limits and descriptions related to the frequency band in this study are shown in Table 1. Since the EMF measured in this study can be considered to be within the far-field zone, only the electric field strength controlling limits need to be concerned, plotted in Fig. 4.

**Table 1:** Limits for public exposure to EMFs of the current standard in China.

Frequency Range	Electric Field Strength *E* (V/m)	Magnetic Field Strength *H* (A/m)	Equivalent Plane Wave Power Density *S*_*eq*_ (W/m^2^)
0.1-3 MHz	40	0.1	4
3-30 MHz	67/*f*^0.5^	0.17/*f*^0.5^	12/*f*
30-3000 MHz	12	0.032	0.4
3000-15000 MHz	0.22*f*^0.5^	0.00059*f*^0.5^	*f*/7500
15-300 GHz	27	0.073	2

*Note:

1. *f* as indicated in the frequency range column.

2. $E^{2}$, $H^{2}$, $S_{eq}$ are to be averaged over any 6-min period.

3. In the far-field region, restrictions could be made on *E*, or *H*, or $S_{eq}$; in the near-field region, restrictions should be made on both *E* and *H*.

**Figure 5:**
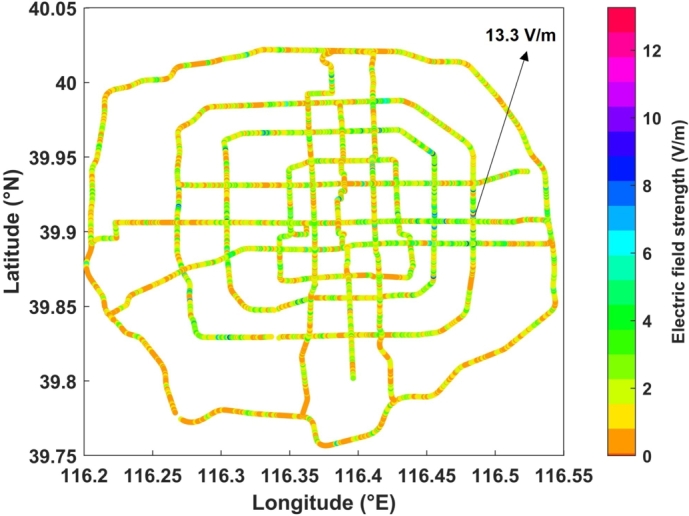
Colorscale map of the environmental EMF data.

**Figure 6:**
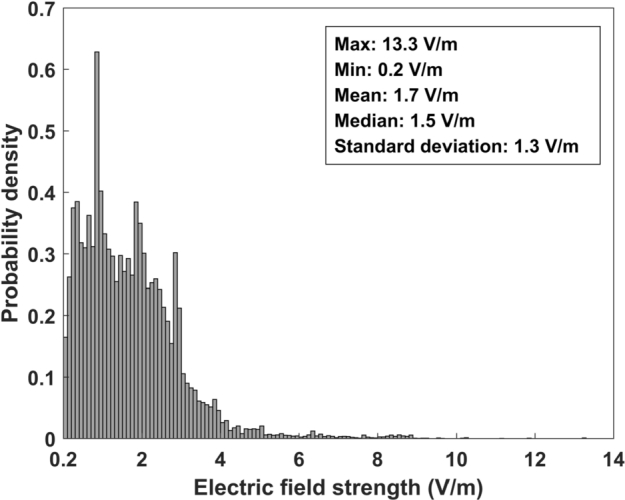
Statistical analysis results of the environmental EMF data.

The EMF data in Section 2.1 are plotted in Fig. 5, where the horizontal and vertical coordinates are longitude and latitude and the color scale indicates the electric field strength. The statistical analysis results are shown in Fig. 6. It can be seen that the electric field strength of most of the sampling points (about 89%) is within 3 V/m, but it is relatively high in some road sections, even exceeding 12 V/m. According to GB 8702-2014, when the public is exposed to EMFs of multiple frequencies, the following relationship should be satisfied between 100 kHz and 300 GHz:

(1) $$\sum_{j=100kHz}^{300GHz}E_{j}^{2}/E_{L,j}^{2}\leq 1$$

where $E_{j}$ is the measured electric field strength (V/m) at frequency *j*, and $E_{L,j}$ is the electric field strength (V/m) at frequency *j* in Table 1. Since the lowest controlling limit corresponding to the measured frequency band is 12 V/m, the sampling points with electric field strength lower than 12 V/m meet the standard; while for those higher than 12 V/m, further spectral analysis is required to determine whether (1) is satisfied, so as to check whether the standard is met.

The case where the electric field strength is higher than 12 V/m is located at approximately 116.483885°E and 39.912138°N (the area marked by arrows in Fig. 5). The center point of the area is selected for further spectrum scanning. For complete coverage of the frequency range 100 kHz to 6.5 GHz, three Schwarzbeck biconical antennas EFS 9218 (9 kHz to 300 MHz), SBA 9113B (80 MHz to 3 GHz), and SBA 9112 (3 GHz to 18 GHz) were used with spectrum analyzer Tektronix RSA 507A for spectrum measurements. For each antenna, it was set on a tripod on the ground at a height of 1.7 m and connected to the analyzer through a coaxial cable. The voltage of RSA 507A $V_{dBuV}(f)$ was converted into electric field readings through the following formula:

(2) $$E_{dBuV/m}(f)=V_{dBuV}(f)+AF_{dB/m}(f)+\alpha_{dB}(f)$$

where $AF_{dB/m}(f)$ is the antenna factor, and $\alpha_{dB}(f)$ is the attenuation of the coaxial cable. The detection method of RSA 507A was set to ‘CAVerage’ mode, which is the CISPR average detection. The resolution bandwidth setting is shown in Table 2 [28], [29]. Combining Table 2 with the frequency band of the antennas, the frequency band of the spectrum measurements is divided into 5 parts: 100 kHz - 30 MHz, 30 MHz - 300 MHz, 300 MHz - 1 GHz, 1 GHz - 3 GHz, 3 GHz - 6.5 GHz. Each part was continuously scanned for not less than 6 min, so that the root-mean-square value of the spectral intensity over 6 min could be obtained. Then (1) could be calculated based on the spectrum scanning data, and the result is

(3) $$\sum_{j=100kHz}^{300GHz}E_{j}^{2}/E_{L,j}^{2}=1.06>1$$

Therefore, it can be concluded that the standard is not met here. The main contribution to the radiation spectrum is the mobile communication base stations. The top five frequency bands with the highest spectral intensity are listed in descending order as shown in Table 3, accounting for about 80% of the overall radiation spectrum. After on-the-spot investigation, the authors found that there are multiple communication base stations in the above-mentioned area, and the total radiation power is relatively large during the peak flow of people, which is the main reason why the public exposure to environmental EMFs does not meet the standard.

**Table 2:** Resolution bandwidth setting for spectrum measurements.

Frequency range	Resolution bandwith
100 kHz - 30 MHz	100 Hz
30 MHz - 1 GHz	1 kHz
1 GHz - 6.5 GHz	10 kHz

**Table 3:** The top five frequency bands with the highest spectral intensity.

	Frequency band	Comprehensive intensity $\sqrt{\sum_{j=f_{1}}^{f_{2}}E_{j}^{2}}$	Ratio to limit values $\sum_{j=f_{1}}^{f_{2}}E_{j}^{2}/E_{L,j}^{2}$
1	TDD Band41 *f*_1_: 2496 - *f*_2_: 2690 MHz	6.35 V/m	0.280
2	FDD Band3 *f*_1_: 1805 - *f*_2_: 1880 MHz	5.45 V/m	0.206
3	FDD Band1 *f*_1_: 2110 - *f*_2_: 2170 MHz	4.86 V/m	0.164
4	TDD Band39 *f*_1_: 1880 - *f*_2_: 1920 MHz	3.98 V/m	0.110
5	TDD Band34 *f*_1_: 2010 - *f*_2_: 2025 MHz	2.43 V/m	0.041

## 4 Association analysis methods

**Figure 7:**
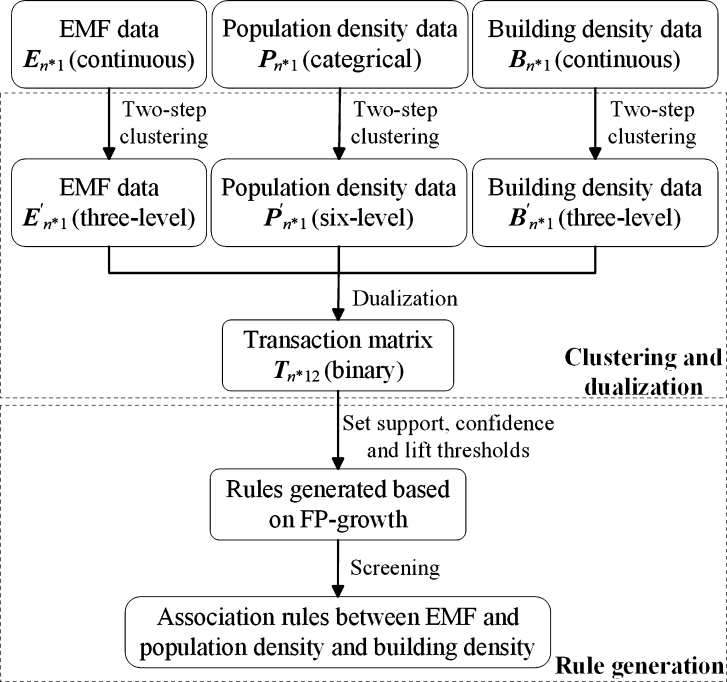
The proposed association rule mining procedures.

It is time-consuming and impractical to measure environmental EMFs in all areas of a city to check whether they meet the standard. It would be helpful to be able to analyze the association between the environmental EMF and other data, so as to quickly obtain the general condition of the environmental EMF in any area. Therefore, this study proposes to mine the association rules between the environmental EMF and population density and building density. An association rule can be expressed as X→Y, which denotes that *Y* is much more likely to occur whenever *X* occurs. The classic algorithms for association rule mining include Apriori [30] and FP-growth [31], but they cannot be directly used to analyze the data in this study because the algorithms deal with binary data (0 and 1). Therefore, firstly, the original data of EMF, population density and building density are classified by two-step clustering method; then the classified data are dualized to generate a transaction matrix that can be processed by the association rule mining algorithm. Next, support, confidence and lift thresholds are set according to the characteristics of the transaction matrix, and rule generation is performed. Finally, the meaningful association rules between the environmental EMF and population density and building density are obtained by rule screening. The above procedures are shown in Fig. 7 and are described in detail in two parts below.

### 4.1 Clustering and dualization

The original data of electric field strength, population density and building density were classified and dualized because they were continuous, categorical and continuous data, respectively, rather than the binary data processed by the association rule mining algorithm. In this paper, the cluster analysis method is used to classify the data in order to make the data of the same class more similar. Commonly used cluster analysis algorithms include K-means [32] and two-step cluster [33]. The two-step cluster method was chosen in the present study because it is the only method that can address both continuous and categorical data. Moreover, the two-step cluster has the ability to handle vast datasets and automatically determine the optimal number of clusters based on statistical criteria [34].

The process of the two-step cluster method, as the name suggests, has two steps termed ‘pre-clustering’ and ‘clustering’. The pre-cluster step uses a sequential clustering approach. It scans the data records one by one and decides if the current record should be merged with the previously formed clusters or starts a new cluster based on the distance criterion:

(4) $$C_{E}=2\sqrt{\frac{1}{JK_{A}}\sum_{j=1}^{J}\sum_{k=1}^{K_{A}}{\overset{ˆ}{\sigma }}_{jk}^{2}},$$

(5) $$C_{L}=\ln(\prod_{k}R_{k}\prod_{k}L_{k}).$$

$C_{E}$ is the criterion for the Euclidean distance, which can only be applied to continuous variables (such as electric field strength and building density). The Euclidean distance between two points is clearly defined. The distance between two clusters is defined by the Euclidean distance between the two cluster centers. $C_{L}$ is the criterion for the log-likelihood distance, which can be applied to both continuous and categorical variables (such as population density grade). The log-likelihood distance between clusters *i* and *j* is related to the decrease in log-likelihood as they are combined into one cluster:

(6) $$d_{L}(i,j)=\xi_{i}+\xi_{j}-\xi_{<i,j>}$$

where

(7) $$\xi_{v}=-N_{v}(\sum_{k=1}^{K_{A}}\frac{1}{2}\ln({\overset{ˆ}{\sigma }}_{k}^{2}+{\overset{ˆ}{\sigma }}_{vk}^{2})+\sum_{k=1}^{K_{B}}{\overset{ˆ}{E}}_{vk}),v=i,j,<i,j>$$

and

(8) $${\overset{ˆ}{E}}_{vk}=-\sum_{l=1}^{L_{k}}\frac{N_{vkl}}{N_{v}}\ln\frac{N_{vkl}}{N_{v}}.$$

In (4)-(8), $K_{A}$ is the number of continuous variables, $K_{B}$ is the number of categorical variables, *J* is the number of continuous variables in cluster *j*, $R_{k}$ is the range of the *k*th continuous variable, $L_{k}$ is the number of categories for the *k*th categorical variable, $N_{v}$ is the number of records in cluster *v*, $N_{vkl}$ is the number of records in cluster *v* which belongs to the *l*th category of the *k*th categorical variable, index <i,j> represents the cluster formed by combining clusters *i* and *j*, $\sigma_{jk}$ is the estimated variance of the *k*th continuous variable in cluster *v*, $\sigma_{k}$ is the estimated variance of the *k*th continuous variable for all records.

The cluster step takes sub-clusters resulting from the pre-cluster step as input and then groups them into the optimal number of clusters. Since the number of sub-clusters is much less than the number of original records, traditional agglomerative hierarchical clustering method can be used effectively. Clusters are recursively merged. The pair of clusters with the smallest distance between them is selected and merged into a single cluster. The process repeats until all clusters have been merged. Because the clusters are merged recursively in this way, it is easy to compare solutions with different numbers of clusters.

**Figure 8:**
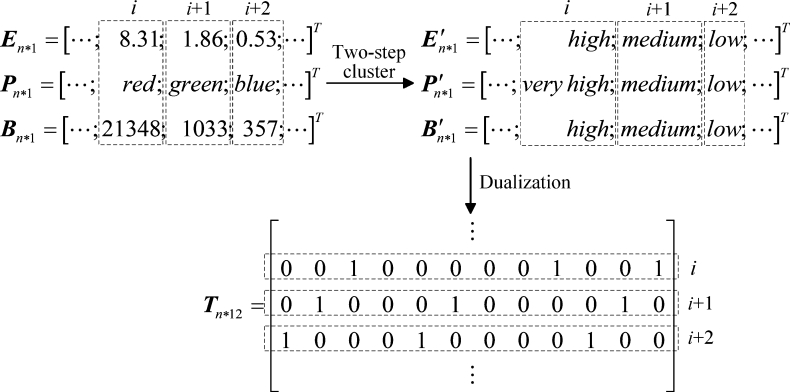
Schematic diagram of the process of forming the transaction matrix from the original data.

The original data of electric field strength, population density, and building density $E_{n⁎1}$, $P_{n⁎1}$, $B_{n⁎1}$ (n=13412) are clustered by the above two steps to form the new hierarchical data $E_{n⁎1}^{\prime }$, $P_{n⁎1}^{\prime }$, $B_{n⁎1}^{\prime }$, and the results are shown in Table 4, Table 5, Table 6. Next, $E_{n⁎1}^{\prime }$, $P_{n⁎1}^{\prime }$ and $B_{n⁎1}^{\prime }$ are dualized to form a transaction matrix that can be processed by the association rule mining algorithm. The elements of the matrix are composed of 0 (representing ‘no’) and 1 (representing ‘yes’). Each row of the matrix represents the data of a sampling point, denoted as a transaction; Each column of the matrix represents a level of the data, denoted as an item, and there are 12 items: $E^{\prime }=low$, $E^{\prime }=medium$, $E^{\prime }=high$, $P^{\prime }=very\;low$, $P^{\prime }=low$, $P^{\prime }=medium$, $P^{\prime }=slightly\;high$, $P^{\prime }=high$, $P^{\prime }=very\;high$, $B^{\prime }=low$, $B^{\prime }=medium$, $B^{\prime }=high$. For example, at the *i*th sampling point, if the electric field strength is high, the population density is very high, and the building density is medium, then the *i*th row of the transaction matrix $T_{n⁎12}$ is [0 0 1 0 0 0 0 0 1 0 1 0]. The above process of clustering, dualization and forming the transaction matrix $T_{n⁎12}$ is shown in Fig. 8 by taking three sampling points as an example.

**Table 4:** The hierarchical value of electric field strength.

Electric field strength (V/m)	Hierarchical value
[0, 1.5)	low
[1.5, 4.0)	medium
[4.0, 13.5)	high

**Table 5:** The hierarchical value of population density.

Population density	Hierarchical value
purple	very low
indigo, blue	low
green	medium
yellow	slightly high
orange	high
red	very high

**Table 6:** The hierarchical value of building density.

Building density (m^2^/*π*300^2^m^2^)	Hierarchical value
[0, 8834)	low
[8834, 31654)	medium
[31654, 99900)	high

### 4.2 Rule generation

After the original data of electric field strength, population density, and building density are converted into binary transaction matrix, classical association rule mining algorithms such as Apriori and FP-growth can be used to generate rules. The FP-growth algorithm is adopted in this study because it uses a compressed representation of the transaction dataset to efficiently generate frequent itemsets, and it is more efficient than Apriori for some datasets.

First, set the support, confidence and lift thresholds of the rule. For a rule X→Y in this study, *X* and *Y* are one or more of the 12 items described in Section 4.1. The support (*s*), confidence (*c*), and lift (*l*) are used to evaluate the frequency of *X* and *Y* appearing in the transaction matrix at the same time, the frequency of *Y* appearing in the transactions that contain *X*, the correlation of *X* and *Y*, respectively. They are calculated as follows:

(9) s(X→Y)=σ(X→Y)/n

(10) c(X→Y)=σ(X→Y)/σ(X)

(11) l(X→Y)=c(X→Y)/s(Y)

where σ(⋅) represents the count, i.e., the number of transactions (sampling points) that contain the itemsets in parentheses. From (9)-(11), it can be derived that the value ranges of support, confidence and lift are [0,min(s(X),s(Y))], [0,1], [0,1/s(Y)]. Half of the maximum value in the value range is set as the threshold in this study, i.e., the support threshold $s_{min}=0.5\cdot min(s(X),s(Y))$, the confidence threshold $c_{min}=0.5$, and the lift threshold $l_{min}=0.5/s(Y)$.

Then, the FP-tree of the sampling point data is constructed. The transaction matrix $T_{n⁎12}$ is scanned to collect the set of frequent items and their support counts, and $T_{n⁎12}$ is sorted in support count descending order denoted as ***D***. The root of the FP-tree is labeled as ‘null’. For each transaction (denoted as $\left[d_{i1}|d_{i}\right]$, where $d_{i1}$ is the first element and $d_{i}$ is the remaining list) in ***D***, call the following procedure denoted as $insert\_tree([d_{i1}|d_{i}],t)$: If *t* has a child *N* the same as $d_{i1}$, then increment *N*'s count by 1; else create a new node *N*, and let its count be 1, its parent link be linked to *t*, and its node-link to the nodes with the same item name via the node-link structure. If $d_{i}$ is nonempty, call $insert\_tree(d_{i},N)$ recursively. So far, the construction of the FP-tree (denoted to as tree) is completed.

Finally, the association rules are mined using the FP-tree of the sampling point data, and the mining process is shown in Table 7. All patterns generated by this process are the complete set of rules, and then each rule is screened according to the factors of interest (e.g., this study focuses on rules containing electric field strength, but not on rules containing only population density and building density) and the support, confidence, and lift thresholds. In this way, valid association rules between electric field strength and population density and building density are obtained.

**Table 7:** Rule mining process.

	Procedure *FP*_*growth*(*tree*,*α*)
1:	**if***tree* contains a single path *P***then**
2:	**for each** combination (denoted as *β*) of the nodes in *P*
3:	generate pattern *β* ∪ *α* with *support*_*count*=
	minimumsupportcountofnodesinβ;
4:	**else for each***a*_*i*_ in the header of *tree* {
5:	generate pattern *β* = *a*_*i*_ ∪ *α* with *support*_*count*=
	*a*_*i*_.*support*_*count*;
6:	construct *β*'s conditional pattern base and then *β*'s
	conditional FP-tree *tree*_*β*_;
7:	**if***tree*_*β*_ ≠ ∅ **then**
8:	call *FP*_*growth*(*tree*_*β*_,*β*);}

## 5 Association rules profiling

The data of electric field strength, population density, and building density of 13412 sampling points in this study were analyzed using the association rule mining procedure in Section 4. The final association rules were screened out as shown in Table 8. The rules in Table 8 are firstly arranged in the order of containing $E^{\prime }=low$ (Rule 1-8), $E^{\prime }=medium$ (Rule 9-11), $E^{\prime }=high$ (Rule 12), and secondly arranged in descending order of confidence. Rule 1 and 8 show that low building density areas tend to be present around sampling points with low-level electric field strength; this tendency is even more pronounced if the case of very low population density is also included. Rule 2-7 show that the electric field strength tends to be at the low level in areas with medium or lower population density or low building density. Rule 9 and 10 show that the electric field strength tends to be at the medium level in areas with very high population density or high building density. Rule 11 and 12 show that very high population density tends to be associated with medium and high-level electric field strength.

**Table 8:** The association rules obtained in this study.

	Rule	Confidence	lift	Support
1	$E^{\prime }=low,P^{\prime }=very\;low\to B^{\prime }=low$	89.7%	2.1	8.0%
2	$P^{\prime }=very\;low,B^{\prime }=low\to E^{\prime }=low$	72.8%	1.5	8.0%
3	$P^{\prime }=very\;low\to E^{\prime }=low$	70.0%	1.4	9.0%
4	*P*′ = *low* → *E*′ = *low*	66.9%	1.4	5.5%
5	$P^{\prime }=very\;low\to E^{\prime }=low,B^{\prime }=low$	62.8%	2.5	8.0%
6	*P*′ = *medium* → *E*′ = *low*	61.2%	1.2	5.5%
7	*B*′ = *low* → *E*′ = *low*	60.1%	1.2	25.2%
8	*E*′ = *low* → *B*′ = *low*	50.7%	1.2	25.2%
9	$P^{\prime }=very\;high\to E^{\prime }=medium$	57.5%	1.2	24.7%
10	*B*′ = *high* → *E*′ = *medium*	56.4%	1.2	16.6%
11	$E^{\prime }=medium\to P^{\prime }=very\;high$	53.2%	1.2	24.7%
12	$E^{\prime }=high\to P^{\prime }=very\;high$	70.9%	1.7	2.7%

The above rules reveal the correlation between environmental electric field and population density and building density on the one hand, and provide important inspiration in terms of monitoring and risk warning of environmental EMF on the other hand. In order to detect the risk of EMF exposure in time, corresponding monitoring work needs to be carried out continuously. However, this would be labor-intensive and impractical, especially if all areas of the city are monitored. We already know from the association rules that high-level environmental electric field tends to occur in areas with very high population density, so sampling points for monitoring can be set mainly in areas with very high population density. In addition, these areas can be further refined. By purchasing the specific data of population density from the corresponding company, the level of very high population density in this study can be further divided into several levels. Then the association rules between these levels and the environmental EMF can be analyzed, so as to more efficiently and pertinently target finer areas for monitoring and risk warning.

## 6 Conclusions

In this study, broadband environmental electric field measurements were carried out on urban roads in Beijing. The electric field strength was within 3 V/m at about 89% sampling points, and was relatively high at a few sampling points. It is noteworthy that the electric field strength at the road section near 116.483885°E and 39.912138°N was 13.3 V/m, which was confirmed to exceed the national standard limit by further spectrum analysis. Such condition has hardly been reported in previous environmental EMF exposure studies.

A set of procedures for mining association rules between the environmental electric field and population density and building density were proposed. The derived rules based on the measurement data of urban roads in Beijing show that the electric field strength tends to be low level (<1.5 V/m) in areas with medium or lower population density (green, blue, indigo, and purple) and areas with low building density (<8834 m^2^/*π*300^2^m^2^); the electric field strength tends to be medium level (1.5-4.0 V/m) in areas with very high population density (red) and areas with high building density (31654-99900 m^2^/*π*300^2^m^2^); while the high-level electric field strength (4.0-13.5 V/m) tends to occur in areas with very high population density.

It is recommended to be alert to the increasing exposure to environmental EMFs due to urban development. Focusing on the monitoring of EMFs in areas with very high population density is an efficient means to detect potential exposure risks in time. Subsequent study can further refine the very-high-level population density into several levels and analyze the associations between these levels and environmental EMF. In addition, other factors (such as real-time traffic conditions, building attributes, etc.) can be added for association rule mining, so as to discover more meaningful associations related to environmental EMFs.

## CRediT authorship contribution statement

Xinwei Song: Conceived and designed the experiments; Performed the experiments; Analyzed and interpreted the data; Contributed reagents, materials, analysis tools or data; Wrote the paper. Mengqi Han: Performed the experiments; Analyzed and interpreted the data; Contributed reagents, materials, analysis tools or data. Yijie Chen: Performed the experiments; Analyzed and interpreted the data. Yuntao Yue: Contributed reagents, materials, analysis tools or data.

## Declaration of Competing Interest

The authors declare that they have no known competing financial interests or personal relationships that could have appeared to influence the work reported in this paper.

## Data Availability

Data will be made available on request.
